# Medical Association of Georgia

**Published:** 1878-07

**Authors:** 


Medical Association of Georgia.—The transactions of the twenty-ninth annual session, held in Atlanta last April, has just been received. We regret the want of time and space to make such notice of the volume as its get-up demands at our hands. While the volume is larger than usual, we are sorry to see that several valuable verbal reports made before the Association have not been prepared and published in the transactions.

